# UK and Ireland survey of MPharm student and staff experiences of mental health curricula, with a focus on Mental Health First Aid

**DOI:** 10.1186/s40545-021-00364-1

**Published:** 2021-08-31

**Authors:** H. C. Gorton, H. Macfarlane, R. Edwards, S. Farid, E. Garner, M. Mahroof, S. Rasul, D. Keating, H. Zaman, J. Scott, I. Maidment, J. Strawbridge

**Affiliations:** 1 Department of Pharmacy, School of Applied Sciences, University of Huddersfield, Queensgate, Huddersfield, HD1 3DH UK; 2 School of Pharmacy, College of Health and Life Sciences, Aston University, Birmingham, UK; 3 Pharmacy Department, Secure and Complex Care, Birmingham and Solihull Mental Health Foundation Trust, Birmingham, UK; 4 Pharmacy Department, Saint John of God Hospital, Stillorgan, County Dublin Ireland; 5 School of Pharmacy and Medical Sciences, Faculty of Life Sciences, University of Bradford, Bradford, UK; 6 Department of Pharmacy and Pharmacology, University of Bath, Bath, UK; 7 School of Pharmacy and Biomolecular Sciences, Royal College of Surgeons in Ireland, Dublin, Ireland

**Keywords:** Mental health, Mental Health First Aid (MHFA), MPharm, Undergraduate

## Abstract

**Background:**

One in four people experience a mental health problem every year and improving mental health care is an international priority. In the course of their work, pharmacists frequently encounter people with mental health problems. The experience of mental health teaching, including Mental Health First Aid (MHFA) training, in undergraduate pharmacy (MPharm) students in the UK and Ireland is not well documented. Students’ viewpoints, contextualised with curricular overviews provided by staff, were analysed to understand their experience.

**Methods:**

An anonymous, online questionnaire was distributed to MPharm students and staff in the UK and Ireland. Students were asked closed questions regarding their course and exposure to MHFA, which were analysed using descriptive statistics. Open questions were included to enable explanations and these data were used to contextualise the quantitative findings. One member of staff from each university was invited to answer a modified staff version of the questionnaire, to provide a curriculum overview and staff perspective.

**Results:**

232 students and 13 staff, from 22 universities, responded. Three-quarters of students did not agree with the statement that ‘mental health was embedded throughout the MPharm’. Most students (80.6%) stated that they were taught neuropharmacology whilst 44.8% stated that their course included communicating with people about their mental health. One-third (33.2%) of students stated that their degree ‘adequately prepared them to help people with their mental health’. Twenty-six students (11.6%) had completed MHFA training of which 89% would endorse inclusion of this within the MPharm. Of those who had not completed the training, 81% expressed a desire to do so. Those who completed MHFA training self-reported greater preparedness than those who did not, but student numbers were small.

**Conclusions:**

Mental health teaching for pharmacy undergraduates is more focussed on theoretical aspects rather than applied skills. MHFA was viewed by students as one way to enhance skill application. The association of the increased self-reported preparedness of those who completed MHFA could be confounded by a positive environmental cultural. MPharm programmes need sufficient focus on real-world skills such as communication and crisis response, to complement the fundamental science.

**Supplementary Information:**

The online version contains supplementary material available at 10.1186/s40545-021-00364-1.

## Background

Improving mental health is a worldwide priority in the World Health Organization (WHO) Mental Health Action plan (2013–2020) [1]. This is echoed in Government and Health policy in the UK and Ireland [2–4]. The National Health Service (NHS) Long Term Plan [3] includes a specific focus on mental health, owing to the considerable morbidity, mortality and socioeconomic impacts of mental health problems. Each year, one-quarter of adults in the UK experience a mental health problem [2], with comparable numbers in Ireland [5]. Given that community pharmacists and their teams are the most commonly visited healthcare professionals [6], community pharmacy could play a significant role in mental health care. Yet, there are no nationally commissioned services to support this [7].

Pharmacists in every sector need to communicate with people with mental health problems. With the exception of mental health specialties, there is a lack of service provision in pharmacy for mental health [7, 8]. It is possible that this is reflected onto priorities in pharmacy curricula. Indeed, shortcomings in mental health education provision have been identified across various healthcare professions [9]. A greater focus on medicines and certain mental health conditions versus broader, social aspects pertinent to mental health was observed in a 2013 survey of pharmacy schools [10]. Conversely, a review of published curricula of 12 pharmacy courses in Australia, 11 staff stated that they included mental health consultation skills and eight considered sociological issues relating to mental health [11].

A patient and caregiver advisory group to The Royal College of Surgeons in Ireland (RCSI) advocated a broad approach to mental health teaching, beyond medication only, when consulted about curriculum redesign [12]. The group’s recommendation that MHFA become a core component in the MPharm has been implemented in RCSI. Founded in Australia in 2000, MHFA is an accredited 8–12 h training course depending on the country-specific version [13]. The purpose of MHFA is to provide mental health awareness, improve confidence in recognising the signs of mental health problems, reduce stigma and focus on initial management of mental health crises, such as suicide and psychosis [13].

In a global systematic review of MHFA training in universities, four studies explored MHFA training in undergraduate pharmacy degrees [9]. One study included 34 pharmacy students in the USA [14] and three studies were based at the University of Sydney, Australia, where MHFA became a compulsory component of the curriculum [15, 16] following a successful controlled trial of voluntary pharmacy student participation in MHFA in 2011 [17]. Students who undertook MHFA training (*n* = 59) were more confident in providing services to people with mental health problems (*p* < 0.01) and displayed fewer stigmatising attitudes (*p* = 0.04) than students who participated in the regular curriculum only (*n* = 258). The extent to which MHFA has been implemented in the UK and Ireland MPharm courses is unknown.

The aim of this research was to establish the attitudes and experiences of pharmacy students in relation to MHFA and specifically to: (i) determine if and how mental health teaching and MHFA is embedded into the MPharm from both student and staff perspectives; (ii) for those who have undertaken MHFA, evaluate students’ experience and application of the training, and for those who have not, assess the desire to participate; (iii) evaluate attitudes and preparedness of MPharm students to respond to mental health problems and whether this influences their engagement with mental health teaching.

## Materials and methods

An online questionnaire was designed and hosted via the Qualtrics platform for 2 weeks in February 2020. All pharmacy students enrolled in an MPharm degree in any pharmacy school in the UK and Ireland (*n* = 32) were eligible to participate. The questionnaire was distributed via a link in an email to a member of pharmacy faculty with good curricula oversight (e.g. MPharm programme leads) in each of the Universities. Suitable members of staff were identified through our networks and through institutional web pages. One member of staff, whether it be the recipient or a nominated alternative, was invited to complete the questionnaire. The purpose of this was to obtain an overview of course content rather than to offer personal views. The member of staff was asked to circulate the questionnaire to all MPharm students in their school. Students were also invited to participate directly through advertisements on social media (e.g. Twitter).

A pilot questionnaire was completed by three third-year MPharm students and one MPharm course leader. Minor edits were made based on the feedback provided. The questionnaire was split based on a filter question to determine if the participant was a staff member or student (Additional file 1).

### Questionnaire

A combination of yes/no/unsure, Likert scale and open-ended questions were included. Questions were developed that related to emerging themes coming from the literature [9, 16, 17, 19]. The initial set of questions related to students’ perception of different aspects of teaching relating to mental health, e.g. pharmacology, patient counselling. Students were then asked if they had participated in MHFA training. If they had, they were asked questions relating to their experience and application and utility of this training. The definition of MHFA was provided and those students who had not undergone this training were asked whether they would be interested in undertaking the training.

Students were then asked a series of questions to determine whether they perceive there to be stigma relating to mental health and their preparedness to support people with their mental health. These questions were loosely based on a previous study which assessed pharmacy students’ confidence after completing suicide prevention training [18]. We summed the answers to preparedness questions to create a ‘sum preparedness’ score (minimum 6, maximum 30).

Staff were asked questions to give an overview of the MPharm curricula in their institution, e.g. ‘Are students given the opportunity to counsel patients on mental health medications?’. In response to feedback from the pilot survey, these were included as open-ended questions for staff so that they could provide more detail and describe examples.

### Ethical approval

This study was approved by the University of Huddersfield School of Applied Sciences Research Integrity and Ethics Committee (SAS-SREIC 11.02.20-1). Support resources were included in the Participant Information Sheet and Debrief Sheet in case reflecting on mental health caused participants distress or discomfort. In both the staff and student questionnaire, the participants were asked to state their School of Pharmacy. This was to collate responses relating to each school. Once data were downloaded, schools were immediately pseudonymised into a code (codes were pre-assigned for all invited schools hence coding exceeds 22).

### Data analysis

Data were analysed using the IBM® SPSS® Statistics Version 26. Descriptive statistics were used to evaluate demographics, questions relating to MHFA experience, experience of each element of Mental Health teaching and students’ perceptions of stigma and central tendency measures of median and interquartile range for preparedness. Parametric and non-parametric comparison statistics were used as appropriate to compare responses based on MHFA-training status.

Qualitative data from open-ended questions were used to contextualise and interrogate the quantitative data. Comments were reviewed and compared to the quantitative findings and illustrative quotes presented alongside relevant quantitative findings.

## Results

**Fig. 1:**
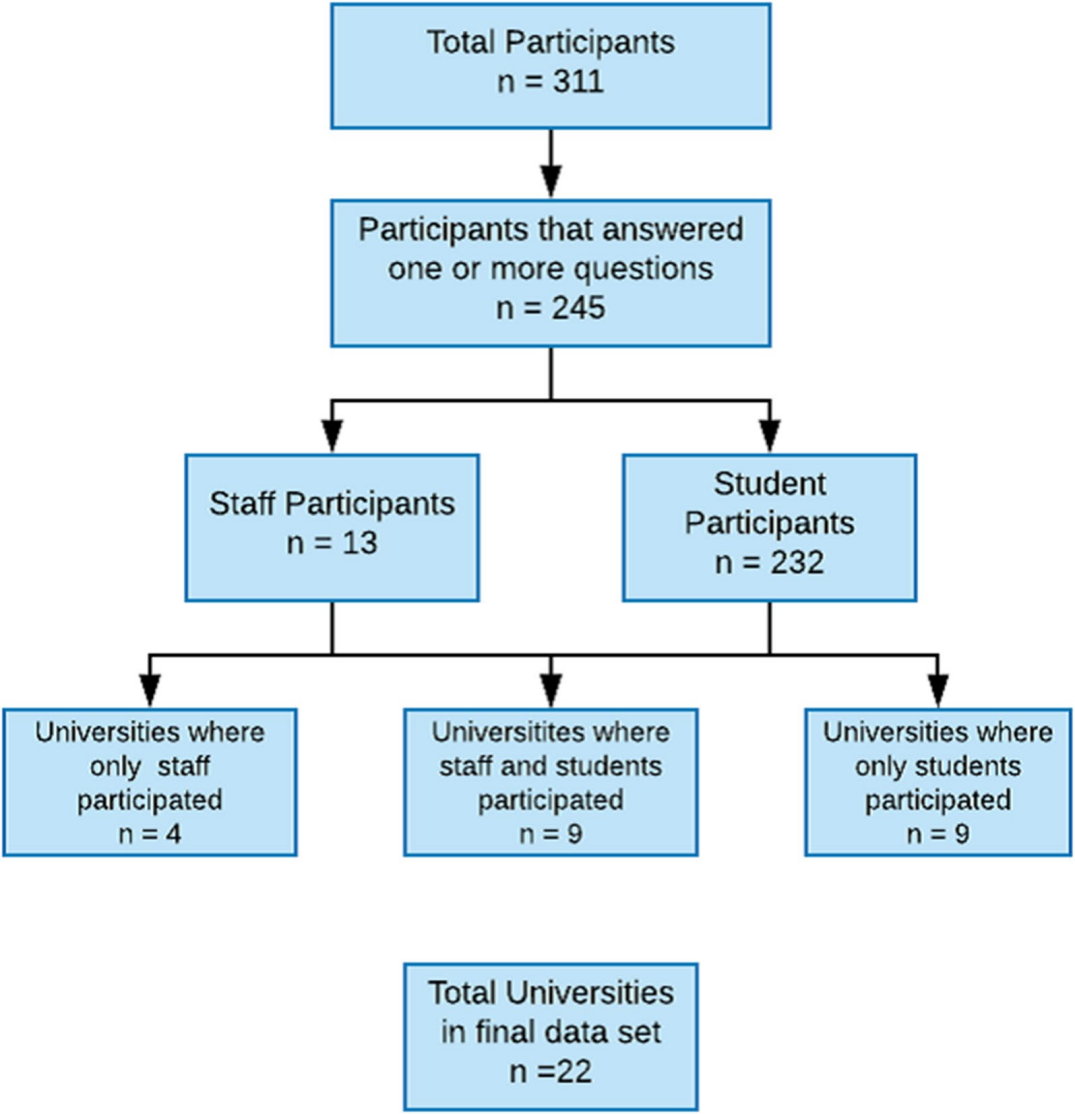
Diagram to show participant inclusion. The number of universities that staff and students contributed from is described, along with overlap, where staff and students from the same institution contributed

Of 311 participants who accessed the survey, 245 answered one or more questions thus were included in the analysis. Thirteen members of staff from individual institutions and 232 students from 18 institutions participated. There were nine institutions where a member of staff and at least one student responded (Fig. 1). The focus was on the results of the student survey, with the staff survey providing curricula overview.

### Demographics

Of 232 student participants, 80% (185) were female, 19% (45) male, one identified as other and one did not respond (Table 1). Most students were in the third (35%) or fourth year (35%) of study.

**Table 1:** Demographics of student participants

Student participants (*n* = 232)	Frequency *n* (%)
*Gender*	
Female	185 (79.7)
Male	45 (19.4)
Other	2 (0.9)
*Year of study*	
1	23 (9.9)
2	45 (19.4)
3	80 (34.5)
4	80 (34.5)
5^a^	4 (1.7)
*University code number*^b^	
2	50 (21.6)
8	5 (2.2)
15	5 (2.2)
18	21 (9.1)
19	25 (10.8)
22	22 (9.5)
23	38 (16.4)
24	11 (4.7)
25	36 (15.5)
Other	19 (8.0)

^a^Some universities have 5 year of study through integrated courses

^b^Randomly allocated code numbers, only universities with 5 or more participants listed individually

### Mental Health First Aid in the curriculum

Twenty-six students (11.2%) reported participating in training that satisfied the MHFA definition. Twenty of these students (77%) attended a single university. Seventy-three percent rated MHFA as beneficial, or very beneficial, and 89% endorsed the idea that MHFA should be mandatory in the MPharm. Of the 205 students who had not completed MHFA (one response invalid), 81% stated that they would like to participate in MHFA training.

Although not all students who participated in MHFA training gave examples of how they had applied training, of those who did, examples from both personal and professional life were given.

“I work part time in a pharmacy and have found it very useful and improved my ability to communicate with patients regarding mental health, and I have applied my training when a patient confided that they were suicidal.” (University 18, 3^rd^ year student).

One participant who had not participated in MHFA training, explicitly recognised its potential value.

“More mental health training definitely needs to be provided within the degree. Perhaps students could be encouraged to attend the Mental Health First Aider course or the uni [university] could ask for a 2-day session to be run on campus—prioritising the healthcare students, e.g. pharmacy & nursing.” (University 23, 3^rd^ year student).

Four of the 13 members of staff stated that MHFA is offered to students. Three were consistent with the definition provided. The official definition of MHFA was provided in the survey, so that participants were clear that MHFA is an official programme that requires completion in its entirety. Yet, one of the participants responded that MHFA was offered then described a shortened, half-day bespoke course, based on MHFA (University 20, staff).

Two staff stated that MHFA was provided by external trainers and in the other, a member of academic was accredited to deliver the training. Some staff felt that aspects were incorporated, but stated that the accredited program was not included “Important component of management and not currently part of our curriculum…parts are touched on but not in a formal way.” (University 2, staff).

Staff offered mixed views on the appropriateness and practicalities of MHFA, in particular cost and target audience. Of those staff who stated MHFA was not currently delivered (*n* = 9), four stated that they would like MHFA to be included, three were uncertain and two thought it was inappropriate.

### Mental health in the curriculum

Students were asked to rate their perception of the inclusion of mental health in the curriculum on a Likert scale from 1 (never) to 5 (constantly embedded). Three-quarters of students selected 3 or below (Table 2). Staff comments illustrated that there is often a modular approach to mental health teaching, although there were some suggestions of integration throughout the curriculum.

“The CNS and mental health module is [are] incorporated in a standalone module in year 2 MPharm degree. It is a big module which spans throughout 2 semesters.” (University 8, staff).

“For example, linked in with diabetes, etc., where the links between physical and mental health and wellbeing are explored.” (University 2, staff).

**Table 2:** Summary of students’ assessment of aspects of mental health teaching and learning that are included in the curriculum

Mental health in curriculum	All (*n* = 232) *N* (%)	Students who declared participation in MHFA (*n* = 26) *N* (%)	Students who did not declare participation in MHFA (*n* = 205)** N (%)	*P* value
*Extent to which taught about mental health in the MPharm*				< 0.001*
1 (never)	20 (8.6)	0	20 (9.9)	
2	67 (28.9)	4 (15.4)	62 (30.5)	
3	89 (38.4)	8 (30.8)	81 (39.9)	
4	40 (17.2)	8 (30.8)	32 (15.8)	
5 (constantly embedded throughout)	14 (6.0)	6 (23.1)	8 (3.9)	
Missing (excluded from comparison)	2 (0.9)	N/A	N/A	
*Agreement with following statements*				
Given opportunity to practise counselling patient on mental health medication	104 (44.8)	24 (92.3)	80 (39.0)	< 0.001*
Clinical check of prescriptions or drug charts relating to mental health medication	130 (56.0)	21 (80.8)	109 (53.2)	0.032*
Taught about how mental health drugs work in the body	194 (83.6)	23 (88.5)	171 (83.4)	0.001*
Learn about laws relating to mental health	118 (50.9)	17 (65.4)	101 (49.3)	0.349
Learning with student mental health nurses	24 (10.3)	1 (3.8)	23 (11.2)	0.583
Placement experience in mental health trust	44 (19.0)	8 (30.8)	36 (17.6)	0.770

* Statistically significant (*p* < 0.05)

** One response invalid so no included in the stratified analysis

The majority of students reported learning about how mental health drugs work in the body (83.6%), experience of clinically checking prescriptions or charts of mental health medication (56%) and learning about laws relating to mental health (50.9%). Forty-five percent of students reported an opportunity to practise mental health medication counselling simulations and some reported learning with mental health nurses (10.3%) or attending a placement at a mental health trust (19.0%). Compared to those who had not undertaken MHFA, students who participated in MHFA were statistically significantly more likely to report opportunities to practice counselling patients on their mental health medication (92.3% vs. 39.0%, *p* < 0.001), clinically check prescriptions for mental health medicines (80.8% vs. 53.2%, *p* = 0.032) and being taught about medication action (88.5%. vs. 83.4%, *p* < 0.001).

In qualitative comments, there was a strong appreciation of the need for enhanced mental health teaching. In particular, parity with that provided for physical health.

“I feel like it could be embedded throughout the programme rather than just focused on in third year and I’m assuming it’ll come up again in fourth year. I also would like more mental health placements as I feel one day in a mental health setting in third year isn’t enough, although I am still grateful to have that one day.” (University 2, 3^rd^ year student).

“We have workshops on physical disease, e.g. hepatic renal and diabetic diseases but little is taught on mental health. I think that there needs to be more focus on the fact many of the physical diseases can actually lead to adverse mental health problems with a focus on treating and managing these problems. As well as teaching on mental health as a whole.” (University 19, 4^th^ year student).

Staff were asked to describe if and how different elements of mental health teaching is incorporated. Of the thirteen, 8 (62%) stated that students practise counselling on mental health medication and that students learn about laws relating to mental health, 10 (77%) described inclusion of clinically checking prescriptions for mental health medication and 11(85%) stated that pharmacology of mental health medication is a focus.

### Perceptions of stigma

When asked their level of agreement with the statement “there is stigma around mental health”, 80.6% students agreed or strongly agreed. There was no statistically significant difference in responses between those who had MHFA training versus those who did not (*p* = 0.713). Qualitative data supported a high level of perceived stigma.

“There is still somewhat of a stigma amongst people on my course and I wish they were a bit more understanding.” (University 19, 3^rd^ year student).

### Preparedness

**Fig. 2:**
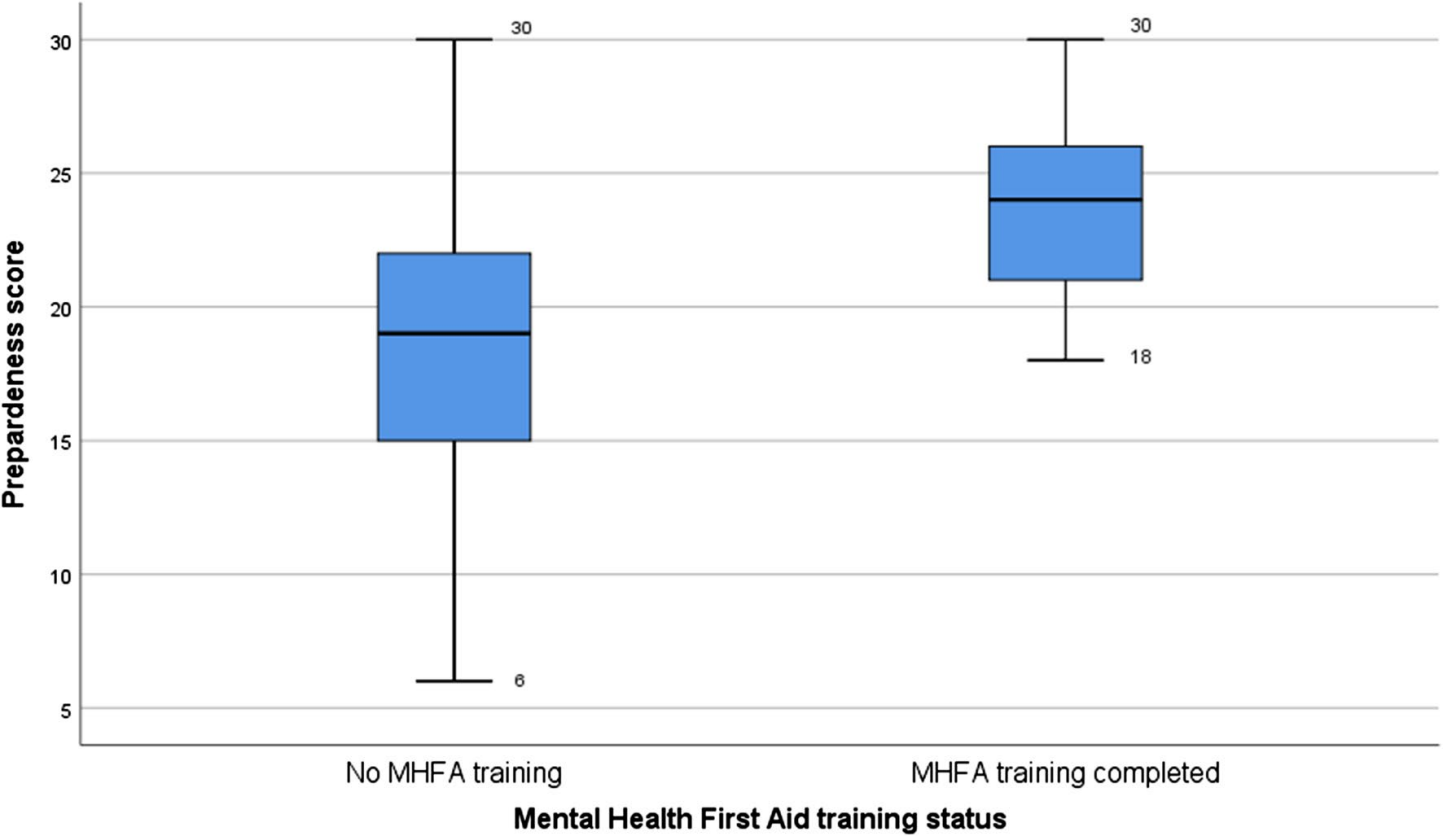
Summed preparedness score displayed separately for those who undertook MHFA training versus those who did not. Preparedness score is a composite of scores (6–30) for individual preparedness statements (each rated from 1—strongly disagree to 5—strongly agree)

The median preparedness score was 19 (IQR 16–23). Those who had MHFA training reported being more prepared (24, IQR 21–27) than those who did not (19, IQR 15–22) (Fig. 2) both for the summative score and at individual statement level (Table 3). The only statement that more than half of students agreed or strongly agreed with (56.2%) was ‘I am confident to talk to people about their mental health’. Just 33.2% of students felt that their degree adequately equipped them to help people with their mental health. Despite some students suggesting adequate preparedness to help people with mental health problems, a strong unmet need was evident in the open comments, specifically relating to communication.

“I guess there is more emphasis surrounding the drugs and how they work and dosages rather than discussing how to talk with someone about their mental health.” (University 18, 4^th^ year student).

“I think we are taught well but learning and practising are completely different worlds, to help make us more confident speaking about mental to people struggling with mental health, instead of awkward or thinking you have to walk on egg-shells with your words, I think more exposure would help, e.g. mental health nurses/students and placements with them, or getting one to talk to us or show us effective mental health consultations.” (University 25, 4^th^ year student).

**Table 3:** Students’ level of agreement with statements regarding their preparedness to respond to mental health

Students’ level of agreement with statements regarding preparedness		All (*n* = 222)** n (%)	Students who declared participation in MHFA (*n* = 25)** n (%)	Students who did not declare participation in MHFA (*n* = 198)** n (%)	*p* value
I feel prepared to identify if someone has a mental health problem	Strongly disagree	6 (2.7)	0	6 (3.0)	0.001*
	Disagree	61 (27.4)	2 (8.0)	59 (29.8)	
	Neutral	63 (28.3)	4 (16.0)	59 (29.8)	
	Agree	77 (34.5)	13 (52.0)	64 (32.3)	
	Strongly agree	16 (7.2)	6 (24.0)	10 (5.1)	
I feel prepared to help somebody with a mental health problem	Strongly disagree	9 (4.0)	0	9 (4.5)	0.013*
	Disagree	55 (24.7)	2 (8.0)	53 (26.8)	
	Neutral	53 (23.8)	3 (12.0)	50 (25.3)	
	Agree	79 (35.4)	14 (56.0)	65 (32.8)	
	Strongly agree	27 (12.1)	6 (24.0)	21 (10.6)	
I am confident to talk to people about their mental health	Strongly disagree	7 (3.1)	0	7 (3.5)	< 0.001*
	Disagree	32 (14.3)	1 (4.0)	31 (15.7)	
	Neutral	52 (23.3)	0	52 (26.3)	
	Agree	107 (48.0)	16 (64.0)	91 (46.0)	
	Strongly agree	25 (11.2)	8 (32.0)	17 (8.6)	
I can confidently counsel a patient on their mental health medication	Strongly disagree	24 (10.8)	0	24 (12.2)	< 0.001*
	Disagree	51 (23.0)	2 (8.0)	49 (24.9)	
	Neutral	38 (17.1)	1 (4.0)	37 (18.8)	
	Agree	79 (35.6)	13 (52.0)	66 (33.5)	
	Strongly agree	30 (13.5)	9 (36.0)	21 (10.7)	
I believe my degree equips me to adequately help people with their mental health	Strongly disagree	13 (5.8)	0	13 (6.6)	< 0.001*
	Disagree	58 (26.0)	0	58 (29.3)	
	Neutral	78 (35.0)	9 (36.0)	69 (34.8)	
	Agree	57 (25.6)	10 (40.0)	474 (23.7)	
	Strongly agree	17 (7.6)	6 (24.0)	11 (5.6)	
I am as prepared to help people with their mental health problems as I am with their physical health problems	Strongly disagree	18 (8.1)	0	18 (9.1)	0.037*
	Disagree	72 (32.3)	4 (16.0)	68 (34.3)	
	Neutral	48 (21.5)	5 (20.0)	43 (21.7)	
	Agree	47 (21.1)	10 (40.0)	37 (18.7)	
	Strongly agree	38 (17.0)	6 (24.0)	32 (16.2)	

^*^Statistically significant (*p* < 0.05)

**Nine people did not answer these questions and are removed from the analysis

## Discussion

This study identified that only 11.2% of student participants had completed MHFA training of which three-quarters were from a single institution. Of those who had completed MHFA, the majority would recommend pharmacy students to undertake MHFA; and of those who did not, most welcomed the opportunity to participate in MHFA. Generally, students did not deem mental health to be fully integrated across the MPharm. There was evidence of focus on neuropharmacology, with less focus on simulated clinical application or communication. Students gave mixed views of self-reported preparedness relating to mental health, with those students having completed MHFA reporting higher levels of preparedness.

### Interpretation

The teaching focus in mental health appears to be towards neuropharmacology or therapeutics, rather than problem solving or communication. This corroborates with a 2013 study of mental health curricula in UK pharmacy schools, where 89% of graduates stated that they did not learn specific communication skills related to mental health in their degree [10]. A small study at a single university reported in 2018 [19] found that 36% of pharmacy graduates were satisfied with university training on mental health. This suggests little progress towards parity of esteem with physical health teaching, in the intervening years. This is likely to result in a disparity in practice. Inadequate coverage of mental health education in curricula for healthcare professionals is an example of structural stigma which needs to be addressed at a leadership level in pharmacy education.

The publication of a Mental Health Competency Framework for Pharmacy Professionals, by Health Education England in 2020, implies that a deficit in mental health exists amongst generalists [20]. Communication is specifically one of the six competency domains described in this Framework. Another domain relates to attitudes and beliefs, thus incorporates stigma. In this study, students indicated a high level of perceived stigma relating to mental health problems, but there was no attempt to measure their personal level of stigma. Perceived stigma is important to understand because it may be a factor in the provision of mental health care by pharmacists even in the presence of positive personal attitudes [21]. Whilst some studies have been quite small, there have been suggestions of a reduction in pharmacy students’ stigma towards mental health owing to MHFA [14], as well as inclusion of a mental health elective [22], and use of contact-based strategies [23, 24]. Integration of MHFA into the pharmacy degree at the University of North Carolina did not influence students’ stigma or attitudes, but did result in increased empathy and self-efficacy, compared to before training [25].

Students who participated in MHFA reported a greater level of preparedness than those who did not, consistent with a recent evaluation of MHFA training involving pharmacists, technicians and students in the USA [26]. In the 12 identified studies of MHFA training of university students of all disciplines, the majority of evaluations were of participants’ self-reported measures [9]. Extensive work in pharmacy students in Sydney has shown that self-reports often overestimated ability to provide MHFA, compared with observed performance [16]. This was especially true in relation to asking about suicide. Furthermore, the value of practising MHFA skills with people with lived experience was identified [27]. These could be important pillars to include in any assessment of the impact of MHFA training, beyond self-reported measures. The initial motivation for roll-out of MHFA in medical and nursing students was a self-care initiative [28]. The potential benefit on self and peers was recognised by students’ qualitative comments in our study, too.

There is a challenge in interpreting the findings, as it is difficult to determine if the benefits for preparedness for practice are attributable to MHFA as an intervention, or the teaching and learning culture that supports the inclusion of MHFA. In one university, for instance, the involvement of patients and carers in curriculum design advocated the inclusion of MHFA in the context of an integrated approach to mental health education. A recent study of medical students indicated that integrating psychiatry in curriculum design decreased stigma and increased understanding of mental health [29]. An integrated model for mental health education in pharmacy was not the most common approach found in the study by Rutter et al., but was seen adopted more in newer schools [10]. These findings suggest that a modular approach is still prevalent. The influence of pharmacists who have previously worked in mental health, or are in patient-facing practice roles, may also have a bearing on curriculum design and the approach to teaching and learning. This was an aspect that Rutter et al. considered and reported that 8 out of 19 Schools of Pharmacy employed pharmacists who had previously worked in mental health [10]. Aspects such as competing priorities and cost considerations likely influence choice to include MHFA, and staff who are strong advocates for mental health may direct choices. Diversity in the culture of mental health education may exist between universities. This will have an impact on the inclusion of MHFA within the context of the overall curricular design and the approach to teaching and learning, and requires further exploration.

The answer to improving mental health education is unlikely to be as simple as to increase content, nor is this practical with busy curricula. Students have identified that the nature of the teaching is important, and that they require more communications training, experiential learning and interprofessional education. The UK Pharmacy regulator [30] requires each of these, but the findings infer that a greater emphasis may be needed, albeit a challenge in the current funding model. Patient involvement in mental health education has been acknowledged to be influential, but has been reported as minimal [10, 31]. All these approaches are likely to be important.

### Strengths

This study was conducted by researchers with a shared interest in mental health from across the UK and Ireland, with experience in education and clinical practice. The multi-centre study obtained the views of students from 13 universities (out of a possible 32) and across multiple years of study offering greater generalisability than studies involving single universities. The input from staff helped contextualise the findings. A considerable amount of qualitative data was generated, which was invaluable for interpretation of the quantitative findings.

### Limitations

The analyses and interpretation of the data have limitations. The total number of students is unknown, and therefore it is not possible to calculate a response rate. Some students may have intended to participate, but missed out due to the short study window (2 weeks) and no reminder. Responses will be influenced by MPharm level, with 1st year students having only 6 months of study. These students would not be placed to comment on the whole curriculum, and level of preparedness for all aspects of practice would be expected to be low. However, over 70% of participants were in year 3 or above, hence they should have had reasonable experience to draw upon. Skewing of students later in the course could have confounded the observed relationship between MHFA training and increased neuropharmacology teaching, as both may occur later in the course but not a consequence of one another.

The comparisons of students who had completed MHFA versus those who have not should be interpreted carefully for two reasons. Firstly, the MHFA group was small (*n* = 26) thus affected available comparisons and associated precision. Secondly, this group was homogeneous, with three-quarters attending a single university. This could conflate the value of MHFA with the environmental culture of the undergraduate degree, as previously discussed.

It was the intention to compare student responses relating to MHFA and curriculum to an overview provided by staff at that institution. This is why only one member of staff per institution was invited. This was not completely possible because data from both parties were only provided for nine institutions, with only a few student responses received from many of them. Instead, staff data have served a purpose to contextualise some of the findings. This has been interpreted with the caveat that staff may present a positive slant.

## Conclusion

This study showed that across respondents from a range of universities in the UK and Ireland, almost 90% of MPharm students have not participated in MHFA training. The inclusion of MHFA in the MPharm was overwhelmingly endorsed by the minority of students who had participated and requested by those who had not. More broadly, research found that teaching was focussed on neuropharmacology rather than the practical skills required for day-to-day clinical practice across all patient-facing sectors. Pharmacy educators and regulators are implored to view their courses holistically so that the emphasis on mental health is proportional to the societal need. Further work to explore pharmacy educators’ experience and views might support this.

### Recommendations

The impact of MHFA training on pharmacy students’ preparedness, subsequent actions and activity; and long-term implication on development and career needs to be measured. MHFA could be one way to the focus on applied skills in mental health. As such, the requirements for first aid training (interpreted as physical first aid) in UK pharmacy training [30] could be widened to include MHFA.

Alongside this, it is imperative that the features that instil a positive environmental culture relating to mental health in the MPharm must be explored. The application of the Health Education England Mental Health Competency Framework to undergraduate curricula should be monitored and evaluated both in terms of process and the skills and knowledge of the resultant pharmacists [20]. Any equivalent guidance in the other nations should be interrogated.

## Supplementary Information

**Additional file 1.** This file contains all of the documentation provided to participants before, during and after participation in the questionnaire. The Participant Information Sheet (PIS) was circulated with study invitation and formed the first page of the questionnaire. The consent items were also circulated and then provided as the second item on the questionnaire. Participants had to read and agree to the items if they wanted to participate, if not, they did not participate. We included a debrief sheet at the end of the questionnaire, to support participants in case of any distress. We include a copy of the questionnaire in the supplementary material. The content was copied verbatim onto the Qualtrics® software which is the software used to deliver the questionnaire to participants.

## Data Availability

The raw data are not available on an independent platform. However, the survey and associated documents are published in the Additional material.
