# Supplementary material for: UK and Ireland survey of MPharm student and staff experiences of mental health curricula, with a focus on Mental Health First Aid
Source: J Pharm Policy Pract. 2021 Aug 31;14:73. doi: 10.1186/s40545-021-00364-1 (PMC8406829; doi:10.1186/s40545-021-00364-1)
Supplement: Supplementary file 1 — Additional file 1. This file contains all of the documentation provided to participants before, during and after participation in the questionnaire. The Participant Information Sheet (PIS) was circulated with study invitation and formed the first page of the questionnaire. The consent items were also circulated and then provided as the second item on the questionnaire. Participants had to read and agree to the items if they wanted to participate, if not, they did not participate. We included a debrief sheet at the end of the questionnaire, to support participants in case of any distress. We include a copy of the questionnaire in the supplementary material. The content was copied verbatim onto the Qualtrics® software which is the software used to deliver the questionnaire to participants. [file 40545_2021_364_MOESM1_ESM.docx]

# Additional Material

Please note that these documents were all online via the Qualtrics platform, so looked slightly different, but content used verbatim.

### Participant information sheet

V3 13/01/2020

[
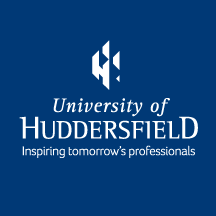
](https://www.google.co.uk/url?sa=i&rct=j&q=&esrc=s&source=images&cd=&cad=rja&uact=8&ved=2ahUKEwjOloXI0-HjAhVIqxoKHXfxBOYQjRx6BAgBEAU&url=/url?sa%3Di%26rct%3Dj%26q%3D%26esrc%3Ds%26source%3Dimages%26cd%3D%26ved%3D%26url%3Dhttps://www.hud.ac.uk/%26psig%3DAOvVaw1Qr26l9Y9ika5MKCr3E6GA%26ust%3D1564748232726186&psig=AOvVaw1Qr26l9Y9ika5MKCr3E6GA&ust=1564748232726186)

**Exploring the views of mental health and Mental Health First Aid (MHFA) training on pharmacy staff and students on the MPharm degree through completion of questionnaire.**

**Participant Information Sheet (PIS)**

Thank you for considering taking part in this research. The aim of this research survey is to understand the approaches and experiences of MPharm academics leaders and students about Mental Health First Aid training (MHFA) in undergraduate pharmacy. Please read this sheet in full before deciding whether to take part. We will ask you to confirm that you have read and understood this sheet and consent statements before you begin the survey. If you have any questions prior to participating in the research, please contact us on the details below

**Who is involved in this research?**

This research is being led by Dr Hayley Gorton, Senior Lecturer in Pharmacy Practice at the University of Huddersfield with third year MPharm students; Sahrrish Farid, Evie Garner, Mohmna Mahroof and Sitherah Rasul.

**Why are we doing this research?**

We want to understand what MPharm leaders and students’ experiences and thoughts on mental health training within the undergraduate pharmacy degree. We have asked you to take part in this research because you are a student or staff member as part of the MPharm degree within the UK or Ireland. We will ask you some questions about your thoughts, feeling and experiences of mental health and mental health first aid training.

**How will I find out about the outcomes of this research?**

We will publish the outcomes of this research in peer reviewed journals and present the work at academic or professional conferences.

The outcomes of the research will be used as part of our 3^rd^ year research projects, contributing to our end of year grade.

**Ethical approval**

This study has been approved by the University of Huddersfield School of Applied Sciences Research Integrity and Ethics Committee (SAS-SREIC 11.02.20-1).

**What does taking part in this research involve?**

You are asked to complete this questionnaire, which should take approximately 10 minutes.

**What happens if I do not want to take part?**

It is your choice whether or not to take part. If you do take part, we will not be able to remove the answers you give from the anonymised data. This is because the data are all anonymised so we would not be able to trace the answers back to you. Although we will ask you which University you attend or work at, this will be coded and anonymised as soon as the data are received. There will be no reference to individual universities by name in any presentation of these data.

**Is my participation confidential?**

Yes. The only identifiable data collected will be the university relating to the staff or students, this will be removed from the raw data and coded meaning it cannot be traced back to the participant or university. The raw data will be saved securely at the University of Huddersfield. Data will be saved for 10 years as per the Code of Practice for Research at the University of Huddersfield. Only summary data will be provided to collaborators at other institutions. There are some questions where you have the opportunity to free-type your answer. We ask you to try and do this without mentioning names of people or places. If you accidentally include these names, we will remove them from the raw data.

**Where can I get more support?**

The topic of mental health can be upsetting. If participating in this survey causes you distress and you wish to stop, you can stop the survey at any time.

If you wish to talk to someone at any point, these support resources may be helpful:

Samaritans: telephone: 116 123; website: [www.samaritans.org/how-we-can-help/contact-samaritan/](http://www.samaritans.org/how-we-can-help/contact-samaritan/)

Mind: telephone open 9am-6pm weekdays only**:** 0300 123 3393; website [www.mind.org.uk/](http://www.mind.org.uk/)

If you are a pharmacist or pharmacy student, you can contact Pharmacist Support; [0808 168 2233](tel:08081682233); website <https://pharmacistsupport.org/>

**How do I contact you?**

If you need to contact us for any reason, including to complain, please contact Dr Hayley Gorton via email: [H.C.Gorton@hud.ac.uk](mailto:H.C.Gorton@hud.ac.uk) or telephone: +44 (0)1484 471491

V3 13/01/2020

### Consent form items

[
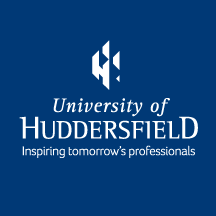
](https://www.google.co.uk/url?sa=i&rct=j&q=&esrc=s&source=images&cd=&cad=rja&uact=8&ved=2ahUKEwjOloXI0-HjAhVIqxoKHXfxBOYQjRx6BAgBEAU&url=/url?sa%3Di%26rct%3Dj%26q%3D%26esrc%3Ds%26source%3Dimages%26cd%3D%26ved%3D%26url%3Dhttps://www.hud.ac.uk/%26psig%3DAOvVaw1Qr26l9Y9ika5MKCr3E6GA%26ust%3D1564748232726186&psig=AOvVaw1Qr26l9Y9ika5MKCr3E6GA&ust=1564748232726186)

**Exploring the views of mental health and Mental Health First Aid (MHFA) training on pharmacy staff and students on the MPharm degree through completion of questionnaire.**

**Consent Form Items**

Please select below if you agree to the following items:

- I have read and understood the Participant Information Sheet.
- I understand that my participation is voluntary.
- I confirm that I am a pharmacy student in the UK or Ireland on an MPharm degree, OR a member of teaching staff on the MPharm degree
- I agree that my data will be used as part of an anonymised dataset and anonymous verbaitm quotes may be used.
- I agree that this data may be used for student reports, academic publications, conference presentations and presented to other audiences in various formats.
- I agree that the data I provide may be used in additional studies where anonymity is maintained.

I agree to take part in this study.

### Debrief sheet

V3 13/01/2020

[
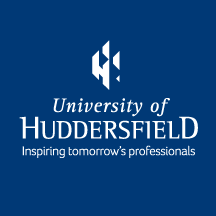
](https://www.google.co.uk/url?sa=i&rct=j&q=&esrc=s&source=images&cd=&cad=rja&uact=8&ved=2ahUKEwjOloXI0-HjAhVIqxoKHXfxBOYQjRx6BAgBEAU&url=/url?sa%3Di%26rct%3Dj%26q%3D%26esrc%3Ds%26source%3Dimages%26cd%3D%26ved%3D%26url%3Dhttps://www.hud.ac.uk/%26psig%3DAOvVaw1Qr26l9Y9ika5MKCr3E6GA%26ust%3D1564748232726186&psig=AOvVaw1Qr26l9Y9ika5MKCr3E6GA&ust=1564748232726186)

**Exploring the views of mental health and Mental Health First Aid (MHFA) training on pharmacy staff and students on the MPharm degree through completion of questionnaire.**

**Debrief Sheet**

Thank you for completing our questionnaire. We know that mental health is an emotive topic and that you may have revisited memories whilst completing this questionnaire. We have produced this debrief sheet so that you have appropriate resources to hand both now and for the future.

**Useful support resources**

**Samaritans:** open 24/7, telephone: 116 123; website: [www.samaritans.org/how-we-can-help/contact-samaritan/](http://www.samaritans.org/how-we-can-help/contact-samaritan/)

**Mind:** open 9am-6pm weekdays only telephone: 0300 123 3393; website: [www.mind.org.uk/](http://www.mind.org.uk/)

**Pharmacist Support:** support for pharmacists. Telephone: [0808 168 2233](tel:08081682233); website: <https://pharmacistsupport.org/>

**Contact the research team**

If you wish to contact the research team, please do so via Dr Hayley Gorton. Email: [H.C.Gorton@hud.ac.uk](mailto:H.C.Gorton@hud.ac.uk); telephone: +44(0) 1484 471491

### Questionnaire

V3, 13/01/2020

**Exploring the views of mental health and Mental Health First Aid (MHFA) training on pharmacy staff and students on the MPharm degree through completion of questionnaire.**

Thank you for your interest in participating in our research about mental health and Mental Health First Aid training.

Please select if you agree to each of the consent statements □

***************************************************************************

1. Which University are you from?

- Aston University
- University of Bath
- University of Birmingham
- University of Bradford
- University of Brighton
- Cardiff University
- University of Central Lancashire
- University College Cork
- De Monfort University
- University of East Anglia
- University of Hertfordshire
- University of Huddersfield
- Keele University
- King’s College London
- Kingston University
- University of Lincoln
- Liverpool John Moores University
- University of Manchester
- Medway School of Pharmacy
- Newcastle University
- University of Nottingham
- University of Portsmouth
- Queens University Belfast
- University of Reading
- Robert Gordon University
- Royal College of Surgeons in Ireland
- University of Strathclyde
- University of Sunderland
- Trinity College of Dublin
- University College London
- Ulster University
- University of Wolverhampton

2. Are you a staff member or student?

Staff or student

**Student**

3. What is your gender?

- Female
- Male
- Other
- Prefer not to say

4. What is your year of study? (5 year option to take into account a sandwich year)

- 1^st^ Year
- 2^nd^ Year
- 3^rd^ Year
- 4^th^ Year
- 5^th^ Year

These questions are to help us to understand how you learn about mental health as part of your undergraduate degree.

5. To what extent are you taught about mental health as part of your degree?

On a scale from 1 to 5, with 1 being never, and 5 being constantly embedded throughout the degree.

- 1 (Never)
- 2
- 3
- 4
- 5 (Constantly embedded throughout the degree)

6. Can you give an overview as to how mental health teaching is incorporated into your degree?

- Free type answer

7. Are you given the opportunity to practice how to counsel patients on mental health medications?

- Yes/ No/ Don’t know

8. Do you clinically check prescriptions or drug charts relating to mental health medications?

- Yes/ No/ Don’t know

9. Are you taught about how mental health drugs work in the body?

- Yes/ No/ Don’t know

10. Do you learn about the laws relating to mental health? (The Mental Health Act 2007 and The Mental Capacity Act 2005)

- Yes/ No/ Don’t know

11. Do you do any learning with student mental health nurses?

- Yes/No/ Don’t know

12. Have you been on placement to a mental health trust?

- Yes/ No/ Don’t know

13. Have you undertaken any Mental Health First Aid training?

- Yes/No/ Don’t know

Mental Health First Aid (MHFA) training in a programme accredited in your country (MHFA England, MHFA Wales, MHFA Scotland, MHFA Northern Ireland and MHFA Republic of Ireland), Thisis a two-day course aiming to raise awareness of mental health knowledge, skills and confidence to recognise signs of mental health.

14. Based on this definition, have you undertaken any Mental Health First Aid training?

- Yes/No/ Don’t know

If Yes,

15. When did you do this the training?

Free type answer

16. Was the training mandatory?

Yes/No/ Don’t know

17.How beneficial did you think the MHFA programme was?

- - On a scale from 1 to 5, 1 being not beneficial at all at 5 being very beneficial

1. Have you applied any of this training in practice e.g. On placements or in pharmacy summer jobs? If yes, please describe.

Free text answer

1. Do you think Mental Health First Aid training should be compulsory as part of the MPharm degree?

Yes/No/ Don’t know

If No,

1. Would you like to participate in Mental Health First Aid training?

Free text answer

1. Why? - free type answer

For these final questions we want to understand your thoughts and feelings on mental health. Please read each statement and select your level of agreement from strongly disagree to strongly agree.

22.

|  | Strongly Disagree | Disagree | Neither Agree nor Disagree | Agree | Strongly Agree |
| --- | --- | --- | --- | --- | --- |
| 1. There is stigma around mental health. |  |  |  |  |  |
| 1. I feel prepared to identify if someone has a mental health problem |  |  |  |  |  |
| 1. feel prepared to help somebody with a mental health problem |  |  |  |  |  |
| 1. I am confident to talk to people about their mental health. |  |  |  |  |  |
| 1. I can confidently counsel a patient on their mental health medication. |  |  |  |  |  |
| 1. I believe my degree equips me to adequately help people with their mental health. |  |  |  |  |  |
| 1. I am as prepared to help people with their mental health problems as I am with their physical problems. |  |  |  |  |  |

23.Do you have any other comments about mental health and pharmacy?

**Staff**

1. Are you the lead for the MPharm Degree?

- Yes
- No, - Please specify job role

We would like to understand the overall scope of mental health teaching on the MPharm degree at your university.

1. Can you give an overview as to how mental health teaching is incorporated into the degree?
   - Free type answer
2. Are students given the opportunity to practice how to counsel patients on mental health medications?
   - Free type answer
3. Do students clinically check prescriptions or drug charts relating to mental health medications?
   - Free type answer
4. Are students taught about how mental health drugs work in the body?
   - Free type answer
5. Do students learn about the laws relating to mental health? (The Mental Health Act 2007 and The Mental Capacity Act 2005)
   - Free type answer
6. Do students do any learning with student mental health nurses?
   - Free type answer
7. Have students been on placement to a mental health trust?
   - Free type answer

Mental Health First Aid (MHFA) training in a programme accredited in your country (MHFA England, MHFA Wales, MHFA Scotland, MHFA Northern Ireland and MHFA Republic of Ireland), This is a two-day course aiming to raise awareness of mental health knowledge, skills and confidence to recognise signs of mental health.

1. Based on the above definition do students undertake any Mental Health First Aid training?
   - Yes/No

If Yes,

1. What year groups do the training?
   - Free type answer
2. Was the training mandatory?
   - Yes/ No
3. Is a member of staff the trainer, or is an external trainer brought in?
   - External
   - Staff trainer
   - Other – please specify
4. How long has Mental Health First Aid training been part of the curriculum?
   - Free type answer
5. How is the Mental Health First Aid training taught? (is It a complete 2-day course, or split up throughout the year?)
   - Free type answer
6. Were there any barriers when implementing the course?
   - Free type answer

If No,

1. Would you want to implement first aid training as part of the curriculum?
   - Yes/no/uncertain
2. Why did you answer that this way?
   - Free type answer
3. Do you have any other comments about mental health teaching and learning in the MPharm degree?
   - Free text answer
